# Pneumatic vitreolysis versus vitrectomy for the treatment of vitreomacular traction syndrome and macular holes: complication analysis and systematic review with meta-analysis of functional outcomes

**DOI:** 10.1186/s40942-023-00472-x

**Published:** 2023-05-22

**Authors:** Miguel A. Quiroz-Reyes, Erick A. Quiroz-Gonzalez, Miguel A. Quiroz-Gonzalez, Virgilio Lima-Gomez

**Affiliations:** 1 Retina Department of Oftalmologia Integral ABC (Nonprofit Medical and Surgical Organization), Which is Affiliated with the Postgraduate Studies Division of the National Autonomous University of Mexico, Av. Paseo de las Palmas 735 Suite 303, Lomas de Chapultepec, 11000 Mexico City, Mexico; 2 Institute of Ophthalmology, Fundacion Conde de Valenciana, (Nonprofit Organization), Which is Affiliated with the Postgraduate Studies Division of the National Autonomous University of Mexico, Av. Chimalpopoca 14. Col. Obrera, 06800 Mexico City, Mexico; 3 Juarez Hospital, Public Assistance Institution (Nonprofit Organization), Av. Politecnico Nacional 5160, Colonia Magdalena de las Salinas, 07760 Mexico City, Mexico

**Keywords:** Vitreomacular traction syndrome, Macular hole, Pneumatic vitreolysis, Ocriplasmin, Pars plana vitrectomy

## Abstract

**Background:**

We conducted a systematic review to compare  the effects of pneumatic vitreolysis (PV), enzymatic vitreolysis (EVL) with ocriplasmin, and pars plana vitrectomy (PPV) on vitreomacular traction (VMT) syndrome and macular holes (MHs) to assess their efficacy as treatment options.

**Methods:**

Databases, including PubMed, ClinicalTrials.gov (www.clinicaltrials.gov), the Cochrane Central Register of Controlled Trials (CENTRAL)—including the Cochrane Eyes and Vision Group Trials Register (*The Cochrane Library* 2013, Issue 2)—, Ovid MEDLINE, and EMBASE (January 2000–October 2022), were searched to identify studies comparing the outcomes of PV versus PPV, PPV versus ocriplasmin and ocriplasmin versus PV. RevMan 5.1 was used for the meta-analysis of the studies.

**Results:**

Among the 89 studies, 79 were considered eligible for qualitative analysis, and 10 quantitative studies were subjected to meta-analysis. PPV resulted in better postoperative visual acuity improvement than ocriplasmin (standardized mean deviation (SMD) = 0.38, 95% CI 0.03–0.73, *p* = 0.0003). PV resulted in no significant difference in visual improvement compared  with  PPV (SMD = − 0.15, 95% CI − 0.47 to 0.16, *p* = 0.35). PPV was significantly more effective in terms of the VMT release rate (risk ratio = 0.48, 95% CI 0.38–0.62, *p* = 0.00001) and MH closure rate (risk ratio = 0.49, 95% CI 0.30–0.81, *p* = 0.006) than ocriplasmin. PV was more effective than ocriplasmin in terms of the VMT release rate (risk ratio = 0.49, 95% CI 0.35–0.70, *p* = 0.0001). Qualitative analysis showed MH closure rates of 46%, 47.8%, and 95% and VMT releases rates of 46%, 68% and 100% after ocriplasmin, PV, and PPV treatments, respectively.  Adverse events and postoperative complications occurring after treatment have also been documented in these studies*.*

**Conclusion:**

PPV appears to be the most promising option for MH closure and VMT release, with fewer serious complications than EVL  or PV. However, given the limited number of studies comparing these treatments, further research is needed to establish the superiority of PPV over the other options.

**Supplementary Information:**

The online version contains supplementary material available at 10.1186/s40942-023-00472-x.

## Background

Vitreomacular traction (VMT) syndrome is caused by incomplete posterior vitreous detachment (PVD)  of  the macula [1]. This unusual macular condition was first reported in 1970 by Reese et al. [2], who  confirmed that traction is caused by incomplete PVD in the macula, leading to decreased visual acuity (VA). Macular traction can be anterior and posterior, as in VMT, which is caused by the persistent attachment of the vitreous in the macular region and ultimately leads to macular hole (MH) formation, macular edema, and limited macular retinal detachment [1]. A diverse range of maculopathies, including MHs, epiretinal membranes (ERMs), and cystoid macular edema (CME), have been associated with VMT syndrome [3, 4]. MHs are associated with VMT syndrome due to traction and schisis that results in foveal tissue distortion, focal CME, and subretinal detachment. These instances might be regarded as manifestations of VMT syndrome that confirm its association with MH formation. The first stage of idiopathic MHs has been frequently reported to be linked to perifoveal vitreous detachment [5, 6].

VMT is classified according to its underlying macular pathology, such as diabetic macular edema (DME), the presence of a full-thickness macular hole (FTMH), an ERM or an adhesion with a specific area diameter (focal ≤ 1500 μm and broad > 1500 μm) [7]. The treatment of VMT varies depending on the patient’s symptoms and the severity of traction. Pars plana vitrectomy (PPV) with ERM peeling and internal limiting membrane (ILM) peeling is the most effective treatment for these cases. However, PPV is considered to be the most difficult and invasive method, with a higher risk of complications [8] such as retinal tears (RTs), cataract formation, and endophthalmitis [9, 10]. Although enzymatic vitreolysis (EVL) using ocriplasmin is another option, it is very costly, often unavailable and has an uncertain efficacy [11]. The Food and Drug Administration approved ocriplasmin in 2012 and introduced it commercially for pharmacological vitreolysis, which is considered a less invasive intervention than PPV [11, 12]. However, the VMT release rate, is only approximately 40% [12] and the success rate of ocriplasmin treatment is 26.5% [13]. Furthermore, it is not the optimal treatment for VMT because it is relatively expensive and can result in side effects such as lens subluxation, transitory visual loss, electroretinogram abnormalities, retinal fractures, ellipsoid zone deformities and dyschromatopsias [14, 15], thus greatly limiting its widespread use. Therefore, highly efficient, cost-effective, and much safer treatment methods for VMT and MHs are under further investigation.

To overcome the limitations of the previous techniques, the pneumatic vitreolysis (PV) technique was first defined in 1995 by Chan et al. [16], who achieved great success in treating stage 1–2 MHs. They reported that 96% of patients developed complete PVD and 57% of stage 2 MHs were closed after receiving a 0.3 cc perfluoropropane (C_3_F_8_) gas injection.  Additional studies [17, 18], reported that 80% of isolated VMT cases resolved with PV treatment. Following these findings, Steinle et al. [19] and Özdemir et al. [20], reported the enhanced effectiveness of PV treatment using the postoperative "drinking bird" maneuver (bobbing the head forward and backward as instructed repeatedly until the VMT is released) and long-acting gases, such as C_3_F_8_. The "drinking bird" maneuver is a postoperative technique used in conjunction with PV treatment to enhance its effectiveness [21]. This technique involves the patient moving their head back and forth to facilitate mixing of the injected gas bubble with the vitreous fluid, thereby improving the chance of successful treatment [22]. During the "drinking bird" maneuver, the patient tilts their head forward, with the chin towards the chest, and then slowly raises their head, maintaining a steady movement until the gas bubble reaches the area of the eye requiring treatment. The maneuver is repeated several times during the day to ensure optimal mixing and distribution of the gas bubble [17, 20, 23]. In recent decades, owing to the popularity of optical coherence tomography (OCT), there has been an increase in interest in this therapeutic method, with  the  main advantages  of  minimal invasiveness, low cost, high efficacy, minimal side effects and easy application [24, 25].

The purpose of this systematic review and meta-analysis was to examine the postoperative functional outcomes and compare the incidence of complications of PPV, ocriplasmin vitreolysis, and PV for the treatment of VMT syndrome and MHs.

## Methodology

### Literature sources and searches

This systematic review and meta-analysis was conducted in accordance with the guidelines of the Preferred Reporting Items for Systematic Reviews and Meta-Analyses (PRISMA) statements [26]. A relevant literature search was conducted using PubMed, EMBASE, MEDLINE, and CINAHL. Moreover, the Clinical Trials.gov and ProQuest Dissertations and Theses databases were searched for studies  on VMT, MHs, PV, ocriplasmin vitreolysis and vitrectomy. The  literature search strategies were designed separately for each database Additional file 1 to locate the most relevant data until 2/5/2023. For MEDLINE and EMBASE, OVID^®^ AutoAlerts were set up to alert authors regarding any pertinent new publications. The Association for Research in Vision and Ophthalmology (https://www.arvo.org) websites were also searched. Conferences held through the American Academy of Ophthalmology (AAO) and the Association for Research in Vision and Ophthalmology (ARVO) were searched for all years available, and the meeting materials of the Canadian Society of Ophthalmology (COS) were searched from 2012 to 2022. The ARVO, AAO and COS searches were conducted until 2/5/2023. The following keywords were used for searching conference abstracts: "vitreomacular traction syndrome", “macular hole” “ocriplasmin”, “vitrectomy” and “macular hole surgery”.

### Inclusion criteria

Studies that investigated  the effects of ocriplasmin or surgery on MHs were included. Clinical trials, comparative studies, and nonrandomized studies including cohort studies and retrospective studies were included. Cohort studies and randomized controlled trials (RCTs) were considered eligible for inclusion if they met the following criteria: (1) the studies included patients who were diagnosed with VMT and/or MHs; and (2) the studies reported the effectiveness of PV, EVL using ocriplasmin and vitrectomy for vitreomacular adhesions (VMAs) release, MH closure, or vision improvement. The  studies were required to have a minimum sample size of 10 eyes. Studies could have been performed in any country. But those   with patients who underwent more than 6 months of follow-up were considered eligible.

### Exclusion criteria

Single case reports, editorials, systematic reviews, meta-analyses, articles describing studies with fewer than 10 participants and articles focused on basic research and nonhuman studies were excluded. Studies solely pertaining to age-related macular degeneration or other diagnoses unrelated to VMT and MHs were excluded. Articles that were published in languages other than English were also excluded.

### Screening and filtering of literature

 Articles retrieved through all the database searches were imported into Covidence.org. Duplicate studies were removed, and systematic screening was conducted by two authors (MAQR and VLG). The titles and abstracts were screened, and KAPPA statistics were computed for each stage of filtering before disputes were resolved. In the event of a disagreement, a third reviewer (EAQG) was requested for arbitration. The complete texts of the eligible studies were then uploaded for full screening. Again, the KAPPA statistics were computed before disputes were resolved. All the studies were extracted after evaluating the following relevant information: (1) general information about the study (purpose, aim and findings); (2) followed methodology (study design, entry criteria, study participant, methods, and follow-up period); (3) visual acuity before and after treatment, or the number of eyes with visual acuity that was not corrected, corrected or worseen after treatment; (4) whether the eyes underwent peeling of the ILM at the time of surgery; and (5) safety outcomes and complications during and after PV, ocriplasmin vitreolysis and vitrectomy. The quality of the literature on the completed list was assessed.

### Data extraction

Data were extracted by a single author (MAQR). The retrieved data included basic information (principal author´s last name, year of publication, sample size, study region, study groups, study design, mean age of the participants, total sample size, percentage of cases with MHs, MH closure rates, pre- and posttreatment interventions, mean MH size and VA, percentage of adverse events, participant characteristics (age and sex), treatment details (dose), and disease characteristics (diameter of VMA, presence of ERM and size of MHs).

### Study quality

Modified Downs and Black checklists were used to assess the quality of the included studies Additional file 2. The following items were evaluated in the remaining studies: reporting, external validity, internal validity (bias), internal validity (confounding), and power. Each study was given a total score of 28 according to the checklist. All studies were included in the analysis because of the limited availability of literature. We also reviewed additional studies reporting external validity, internal validity (bias), internal validity (confounding), and power. Each study received a final score of 28 out of a total possible score. All studies were included in the analysis  because of the limited amount of literature that was available.

### Statistical analysis

The meta-analysis was conducted using STATA v. 15.0 (STATA Corporation, College Station, TX, USA). The mean and standard deviation (SD) of both pre- and postoperative VMT and MH parameters were the main outcomes of interest. Regarding the treatment effect, the standardized mean difference (SMD) was calculated by dividing the difference between the mean pre- and postoperative values for each outcome measure (such as MH size and VA) by the SD of the corresponding outcome measure's SD. Each SMD was assigned a weight based on the inverse of its variance, and an average was then calculated. Heterogeneity between studies was computed  using the *I*^2^-test, Z, and χ^2^ statistics. An *I*^2^ statistic > 50% was considered to represent significantly high heterogeneity. Furthermore, a low p-value (< 0.01), a high Z value, and a large χ^2^ value were considered to indicate substantial heterogeneity; therefore, by using the DerSimonian and Laird methods, a random-effects model was applied. Because the data were clinically heterogeneous by nature, random-effects models were applied in each meta-analysis. Forest plots were also generated, and funnel plots were generated to check for publication bias.

## Results

### Search results

**Fig. 1:**
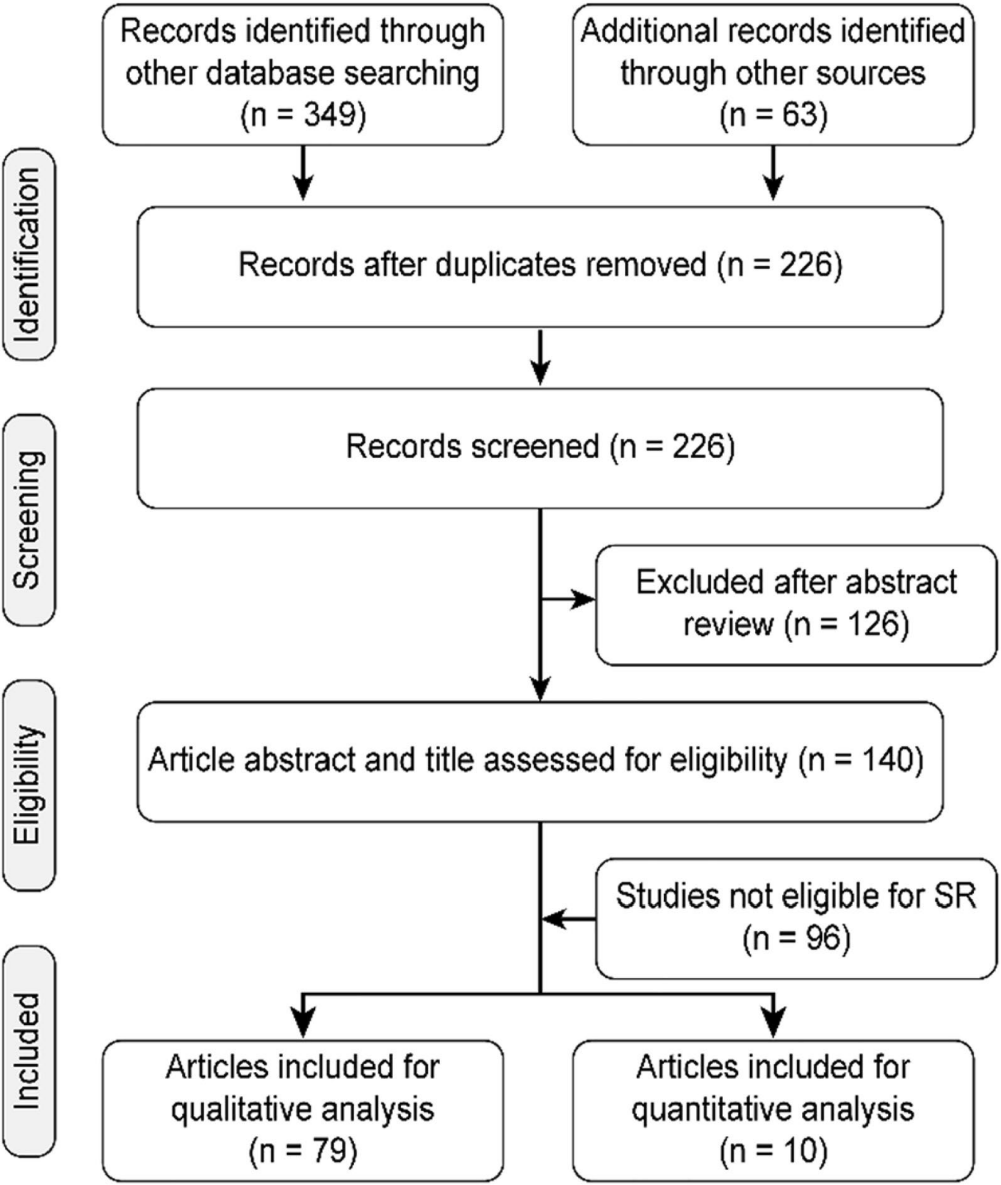
Prisma flow chart showing the detailed search strategy for desired study selection

The database search yielded 412 relevant studies after keyword searches. Reviews, case reports, correspondences, abstracts, and other irrelevant documents were excluded first. After creening the titles and abstracts, 126 additional studies were excluded. Among the remaining studies, 75 studies were  excluded because of insufficient data and irrelevant interventions. Finally, 44 studies were considered eligible for qualitative analysis, and 10 studies were considered eligible for quantitative analysis by assessing the full text (Fig. 1). Among these, we selected 10 different comparative studies: 3 studies compared ocriplasmin versus PV, 2 studies compared PV and PPV, and 5 studies compared PPV with ocriplasmin (Table 1). All the eligible selected studies were comparative nonrandomized, prospective, or retrospective studies. Noncomparative case series, retrospective case series, retrospective analysis, retrospective monocentric analysis, and prospective interventional case series were also included for qualitative and pooled event data analysis.

**Table 1:** Characteristics of all the studies included in the meta-analysis

Author	Study design	Treatment	Quantity	No. of eyes	Mean age (SD)	Mean BCVA (preintervention)	Mean BCVA (post-intervention)	No. of macular holes	Success rate of macular hole closure	Follow-up period	Total no. of diagnosed VMT cases	VMT release
Kumar et al. [29]	Prospective	PV	–	15	63.8 ± 8.38	0.80 ± 0.26	0.70 ± 0.49 (20/100 SE)	15	4 (27%)	3 months	15	12 (80%)
		PPV	–	15	68.33 ± 8.19	0.904 ± 0.44	0.47 ± 0.26 (20/59 SE)	15	15 (100%)	3 months	15	15 (100%)
Alreshaid et al. [30]	Retrospective	PPV	–	11	Not reported	0.81 ± 0.24	0.64 ± 0.26	–	–	10.27 ± 4.63 months	11	11 (100%)
		Ocriplasmin	–	8	Not reported	0.53 ± 0.29	0.52 ± 0.29	N/A	N/A	3.87 ± 2.66	8	5 (62.5%)
Anderson et al. [21]	Prospective	PPV	–	5	64.50	0.72 ± 0.34	0.39 ± 0.20	5	5 (100%)	5.5 months	–	–
		Ocriplasmin	–	8		0.68 ± 0.44	0.27 ± 0.16	8	3 (38%)	6 months	–	–
Greven et al. [31]	Retrospective review	Ocriplasmin	–	51	66.9 (19.1)	0.71 ± 0.35	0.83 ± 0.35	–	–	1, 3 and 6 months	51	22 (43.1%)
		PPV	–	22	69.2 (9.8)	0.62 ± 0.31	0.40 ± 0.23	–	–	1, 3 and 6 months	22	22 (100%)
Juncal et al. [32]	Retrospective case series	Ocriplasmin	0.125 mg in 0.1 ml	11	68.3 ± 8.74	0.56 (20/72 ± 0.28)	0.28 (20/38 ± 0.16	11	4 (36.4%)	12 months	11	4
		PPV	N/A	11	67.8 ± 8.65	0.85 (20/140 ± 0.34)	0.37 (20/47 ± 0.22)	11	10 (90.9%)	12 months	11	10
Nambiar et al. [33]	Retrospective review	Ocriplasmin	0.125 mg (0.1 mL)	17	–	–	–	17	7	4 weeks	17	3 (17.6%)
		PV	0.3 cc of 100% C_3_F_8_	8	–	–	–	8	4 (50%)		8	7 (87.5%)
Scholz et al. [34]	Retrospective case series	Ocriplasmin	125 μg	14	73 ± 10	82 ± 4	81 ± 6	1	1 (100%)	4 months	13	7 (50%)
		PPV	–	10	68 ± 9	78 ± 4	80 ± 5	5	5 (100%)		5	5 (100%)
Steinle et al. [35]	Retrospective	Ocriplasmin	0.125 mg	23	–	–	–	–	–	–	23	48% (11/23)
		PV	0.3 cc of 100% C_3_F_8_ gas	32	–	–	–	–	–	–	32	84% (27/32)
Atkins et al. [28]	Retrospective	Ocriplasmin	0.125 mg	10	–	–	10.34	–	–	1 month	10	50% (5)
		PV	0.3 mL of C_3_F_8_ gas	10	–	–	0.44	–	–	1 month	10	80% (8)
Yao et al. [36]	Prospective	PPV		87	57.78 ± 10.16	1.66	0.5 ± 0.32	–	–	12 months	87	59.8% (52)

BCVA, best-corrected visual acuity; N/A, not applicable; PPV, pars plana vitrectomy; PV, pneumatic vitreolysis; SD, standard deviation

### Characteristics of included studies

The present study provides a comprehensive summary of ten relevant investigations, and the characteristics of each study are outlined in Table 1. The included studies comprised six randomized controlled trials (RCTs), three retrospective analyses, three prospective studies, and two retrospective reviews with retrospective case series. Our analysis primarily focuses on the reported adverse events and complications associated with ocriplasmin, PV, and PPV, which are further presented in Table 2. The data compiled in this study are expected to provide a valuable resource for clinicians and researchers alike for the development of optimal management strategies for these ocular conditions.

**Table 2:** Postoperative complications and adverse events that occurred after different treatments

Adverse events and various complications reported		
Study	Intervention	Postoperative complications (% of cases)
Benz et al. [37]	Ocriplasmin	Cataracts or lens changes (16%), RT without RD (12%), intraoperative/postoperative RD (2%)
Coskey et al. [38]	Ocriplasmin	Worsening of anatomy/vision (25%), developed FTMH (9.6%)
Dihowm et al. [39]	PPV	RD (2.8%), cataract progression/formation (67.6%)
Dugel et al. [40]	Ocriplasmin	Vitreous floaters (37.7%), photopsia (29.5%), color vision test abnormal (28.8%), ophthalmological examination abnormal (19.9%), blurred vision (18.5%)
Han et al. [41]	PV	One eye had localized RD 2 months after surgery
Feng et al. [42]	Ocriplasmin	Development of SFL (33%), post injection EZD (33%), FTMH base enlargement (94%), photopsia (94%), dyschromatopsia (18%), visual blurring (49%)
Kaiser et al. [43]	Ocriplasmin	Vitreous floaters (17.6%), conjunctival hemorrhage (14.6%), eye pain (13.3%), photopsia (12.0%), RT (0.2%), RD (2.4%), retinal edema (5.4%), macular edema (4.1%), increased IOP (3.9%), cataract (2.6%)
Hejsek et al. [44]	25G PPV	Rhegmatogenous RD (3.3%), cataract (0.2%)
Juncal et al. [32]	23 or 25-gauge PPV	EZD (100%), outer segment reflectivity changes (90.9)
	Ocriplasmin	EZD (81.8%), outer segment reflectivity changes (63.6%)
Lim et al. [45]	Ocriplasmin	Photopsia (15%), developed MH (5%), RT (1.4%), RD (1.9%), retinal pigment, epithelium changes (2.9%)
Muqit et al. [46]	Ocriplasmin	No adverse events reported
Nudleman et al. [47]	Ocriplasmin	SRF (73%), EZ changes (56%)
Quezada-Ruiz et al. [48]	Ocriplasmin	Changes in outer band reflectivity (43.47%)
Schumann et al. [49]	Ocriplasmin	SRF (30.5%), cystoid macular edema (6.1%), RD (4.9%), lamellar macular defect (1.2%)
Sharma et al. [50]	Ocriplasmin	EZ changes (47%), reopening of MH (2.9%)
Stalmans et al. [11]	Ocriplasmin	Vitreous floaters (16.8%), photopsia (11.8%), conjunctival hemorrhage (14.6%), injection-related eye pain (13.5%), blurred vision (8.6%), visual impairment (5.4%), increased IOP (3.9%), RT (1.3%), cataract (5.6%), MH (5.2%), RD (0.4%), reduced VA (0.6%)
Stalmans et al. [51]	Ocriplasmin	Drug ineffective (8.5%), vitreous floaters (7.4%), photopsia (7.4%), reduced VA (5.3%)
Willekens et al. [52]	Ocriplasmin	RD (2.6%), SRF (36.8%)
Kumar et al. [29]	PV	In the PV group, 26.66% (4/15) of eyes had a FTMH. Seven eyes required reoperation (four for FTMH and three for unresolved VMT). The PPV group had complications that required reoperation. No endophthalmitis, cataract progression, lenticular dislocation, zonular dehiscence, or uncontrollable increase in intraocular pressure was encountered in either group
	*PPV*	
Primavera et al. [53]	PV	No serious complications were observed
Čokl et al. [54]	PV	Peripheral RT with localized RD in one eye and a small FTMH with a diameter of 220 microns in another eye were observed one week after C_3_F_8_ injection. After one month, another eye with a MH of 330 microns was found in this group (complication rate: 3/29 eyes, 10.3%). A small MH with a diameter of 225 microns was also found in one eye from the SF_6_ group at the one-week follow-up (1/28 eyes, 3.6%)
Wickens et al. [55]	PPV	No serious complications were observed
Alreshaid [30]	PPV	One patient had a lamellar MH after PPV, and 1 patient had a worse BCVA after ocriplasmin injection
	*Ocriplasmin*	
Anderson et al. [56]	Ocriplasmin	Complications, including transient loss of vision, transient disruption of the EZ or subfoveal lucency on OCT, increased MH base diameter, and electroretinographic abnormalities, were observed
	PPV	No serious complications were observed
Greven et al. [31]	Ocriplasmin	Rhegmatogenous RD (4.3%), PVR (2.1%), intraoperative RT (4.3%), PVR detachment (2.1%) intraoperative RT (5.0%). No eyes in the PPV only group developed a rhegmatogenous RD
	*PPV*	
Nambiar et al. [33]	Ocriplasmin	The need for subsequent vitrectomy was lower in the PV group. Novel PV treatment appears to be a more effective and inexpensive option than EVL in this cohort of patients, with fewer patients requiring subsequent vitrectomy
	*PPV*	
Sharma et al. [50]	Ocriplasmin	One eye with a FTMH underwent pharmacologic closure, but then reopened after 2 years. There were no cases of permanent visual loss in this series
Steel et al. [57]	Ocriplasmin	Photopsia (9.8%) and vitreous floaters (6.8%) were the most frequent adverse events
Steinle et al. [35]	Ocriplasmin	EVL with had a lower success rate than C_3_F_8_, and IVO showed significant ORB changes on SD-OCT. Thus, a C_3_F_8_ intravitreal injection appeared to be a safe, inexpensive, and effective option for the treatment of VMT
	*PV*	
Zandi et al. [58]	Ocriplasmin	No serious complications were observed
Baumann et al. [59]	PV	RD occurred in 4 of 47 (8.5%) eyes of the total cohort within a 4-week period, and MHs formed in 4/33 (12.1%) eyes
Fouad et al. [23]	PV	One eye had a RT after PV at upper nasal retina that resulted from vitreous hemorrhage after two weeks of injections
Özdemir et al. [20]	PV	One of 13 eyes had a post procedural RT, and 1 patient had gas migration to the anterior chamber

BCVA, best-corrected visual acuity; C_3_F_8_, perfluoropropane; EZ, ellipsoid zone; EZD, ellipsoid zone deformities; EVL, enzymatic vitreolysis; FTMH, full-thickness macular hole; IOL, intraocular pressure; IVO, intravitreal ocriplasmin; PPV, pars plana vitrectomy; ORB, outer retinal band; PV, pneumatic vitreolysis; PVR, proliferative vitreoretinopathy; MH, macular hole; RD, retinal detachment; RT, retinal tear; SF_6_, sulfur hexafluoride; SFL, subfoveal lucency; SRF, subretinal fluid; SD-OCT, spectral domain-optical coherence tomography; VA, visual acuity; VMT, vitreomacular traction

### Methodological completeness ensured by modified downs and black checklist

To ensure methodological completeness, the quality of all studies included in this analysis was assessed using a modified version of the Downs and Black checklist. The evaluation criteria  were reporting, external validity, internal validity (bias), internal validity (confounding), and power. The quality scores for each study were calculated from a total possible score of 28, with a median score was found to be 15.5. All included studies were analyzed despite variations in quality scores, as the literature available on the topic was limited. A summary of the quality assessment results  is presented in Additional file 2: Table S1.

### Publication bias

**Fig. 2:**
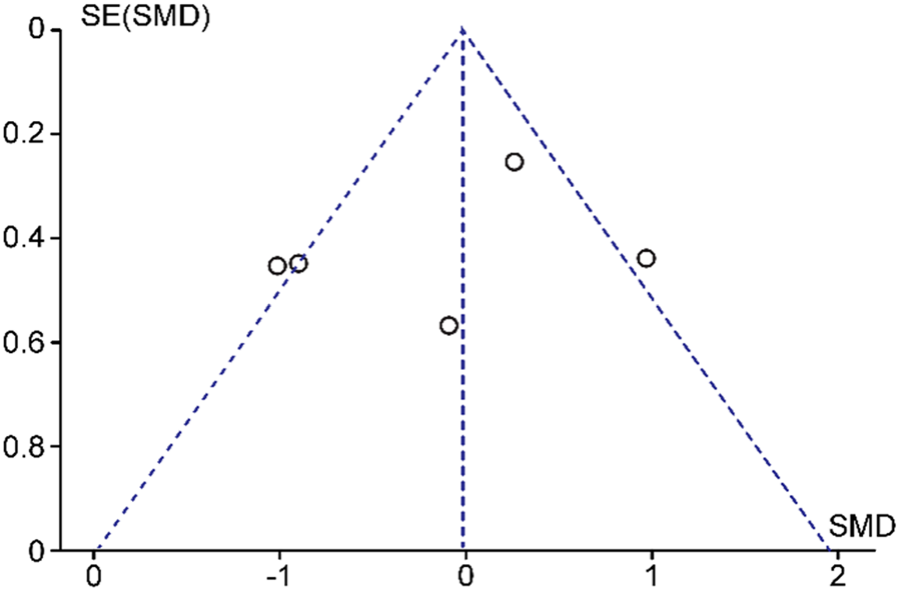
Funnel plot of studies comparing preoperative best-corrected visual acuity (BCVA) of ocriplasmin versus PPV

**Fig. 3:**
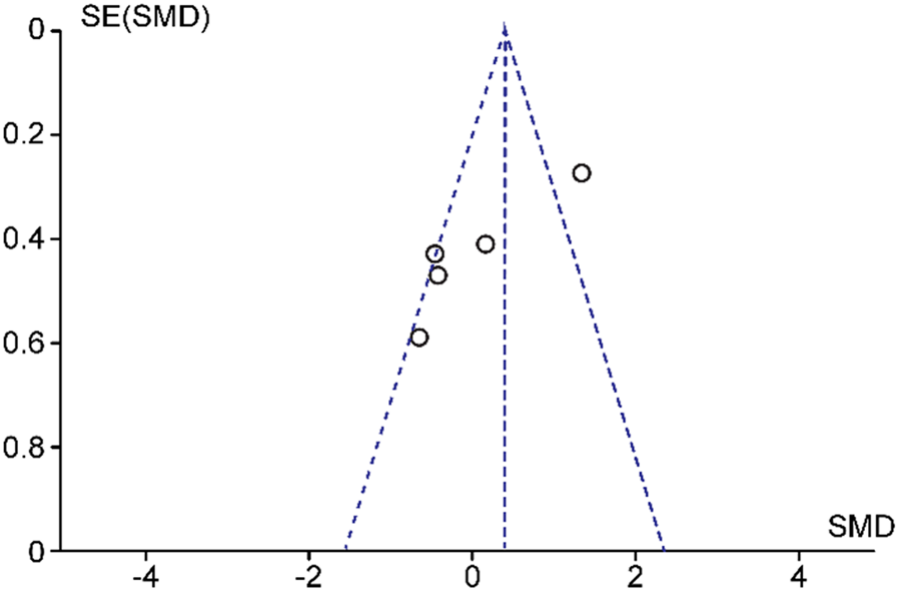
Funnel plot of studies comparing postoperative VA of ocriplasmin versus PPV

**Fig. 4:**
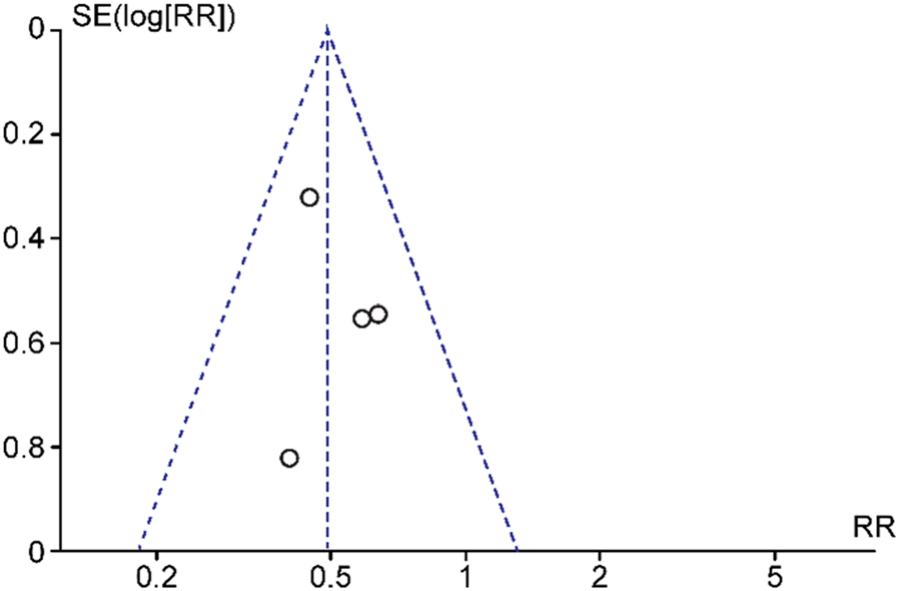
Funnel plot of included studies evaluating the VMT release rate

The funnel plots are scatter plots comparing the estimated intervention effect from each study against a measure of each study size or precision. The funnel plot for preoperative VA (Fig. 2) showed that only 2 studies were located outside the funnel shape, while the funnel plot for postoperative VA showed that only one study was located outside the funnel plot (Fig. 3). As shown in Fig. 4, none of the studies evaluating the different interventions for VMT release and MH closure showed evidence of publication bias.

### Visual outcome efficacy analysis

**Fig. 5:**
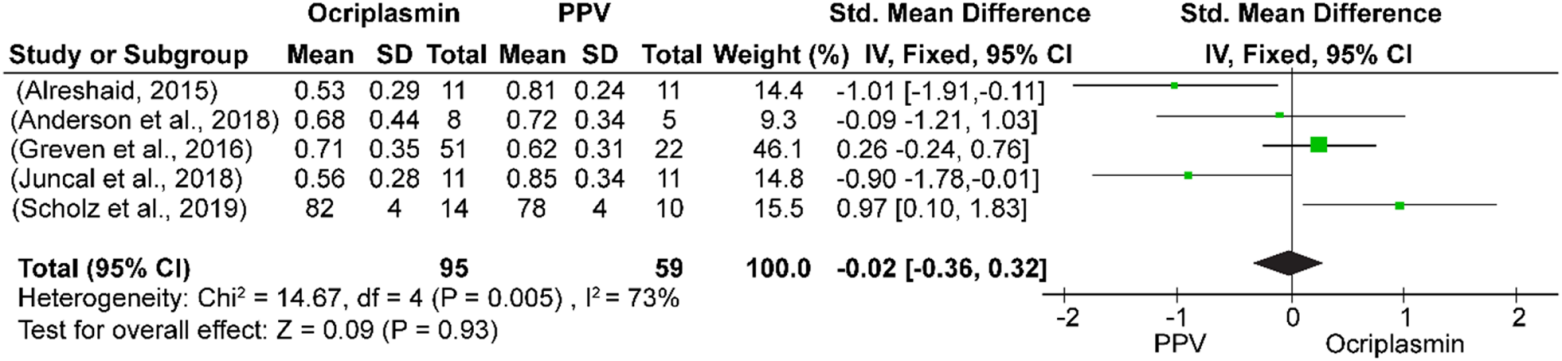
Forest plot of preoperative BCVA of ocriplasmin versus PPV

**Fig. 6:**
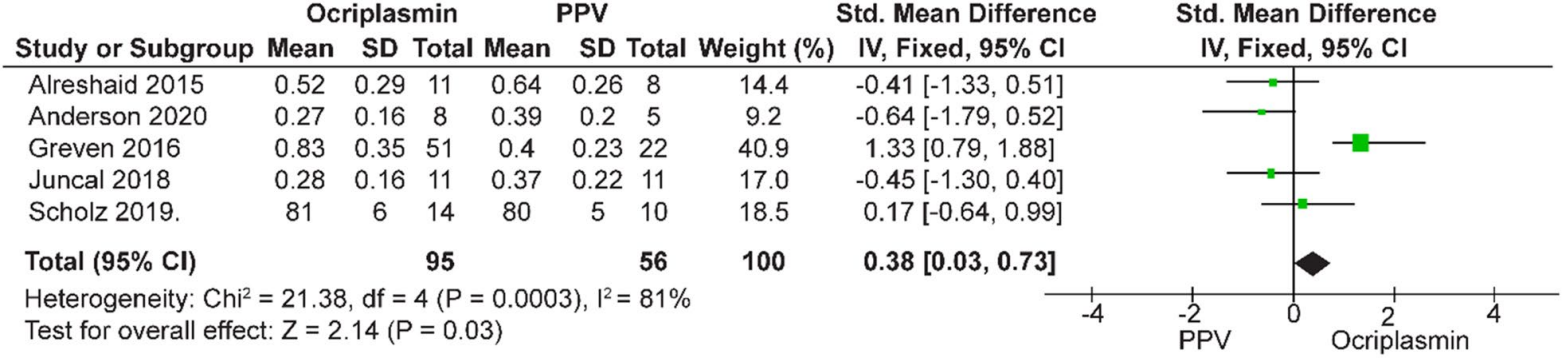
The postoperative BCVA of ocriplasmin versus PPV

**Fig. 7:**
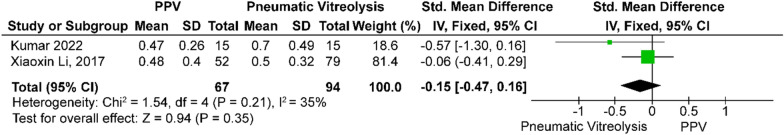
Forest plot of postoperative BCVA of PV versus PPV

In this meta-analysis, three different interventions for VMT release and MH treatment were compared to identify the best treatment for improving VA with fewer complications. Among these interventions, ocriplasmin was compared with PPV and PPV was compared with PV. The analysis of pre- and postoperative VA showed significantly greater improvement after PPV than after ocriplasmin treatment (SMD = − 0.02, 95% CI − 0.36–0.32, *p* = 0.93 to SMD = 0.38, 95% CI 0.03–0.73, *p* = 0.0003) (Figs. 5, 6).  Testing for heterogeneity  revealed a high rate of heterogeneity. Moreover, the postoperative VA improvement was greater in patients who underwent PV (SMD = − 0.15, 95% CI − 0.47 to 0.16, *p* = 0.35) than in those who underwent PPV, but there was no significant difference in the postoperative BCVA (Fig. 7). The comparative study of ocriplasmin and PV [27, 28] did not report the preoperative and postoperative VA, thus an analysis was not conducted.

### Rates of successful VMT release and MH closure

**Fig. 8:**
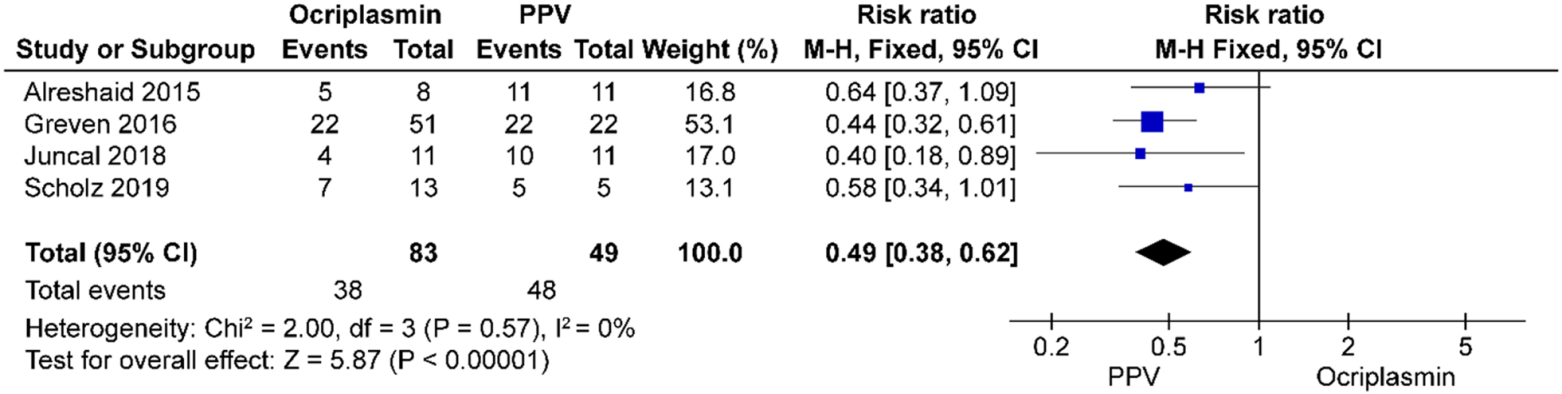
Success rate of ocriplasmin and PPV treatment for VMT release

**Fig. 9:**
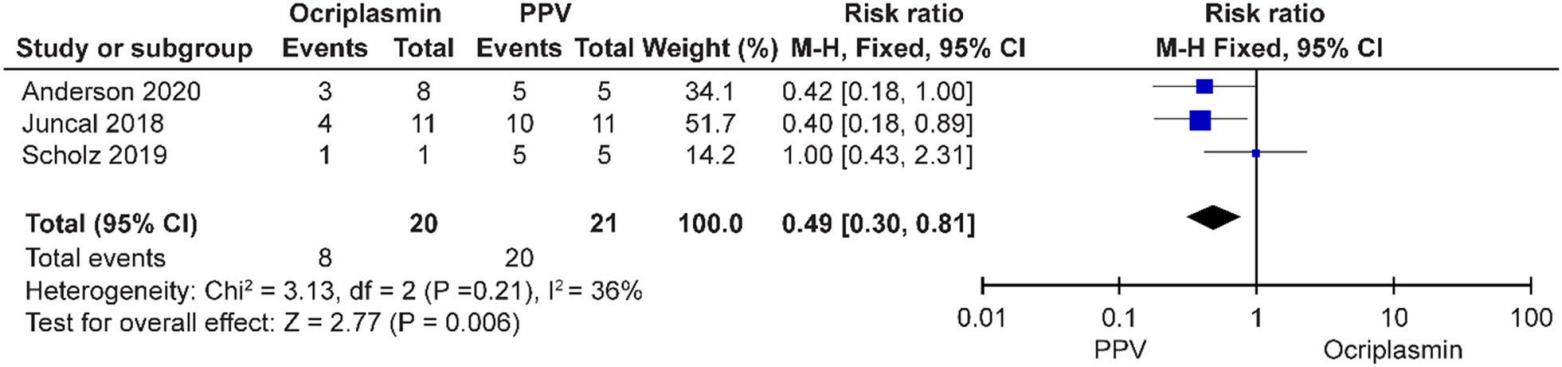
Success rate of ocriplasmin and PPV for MH closure

**Fig. 10:**
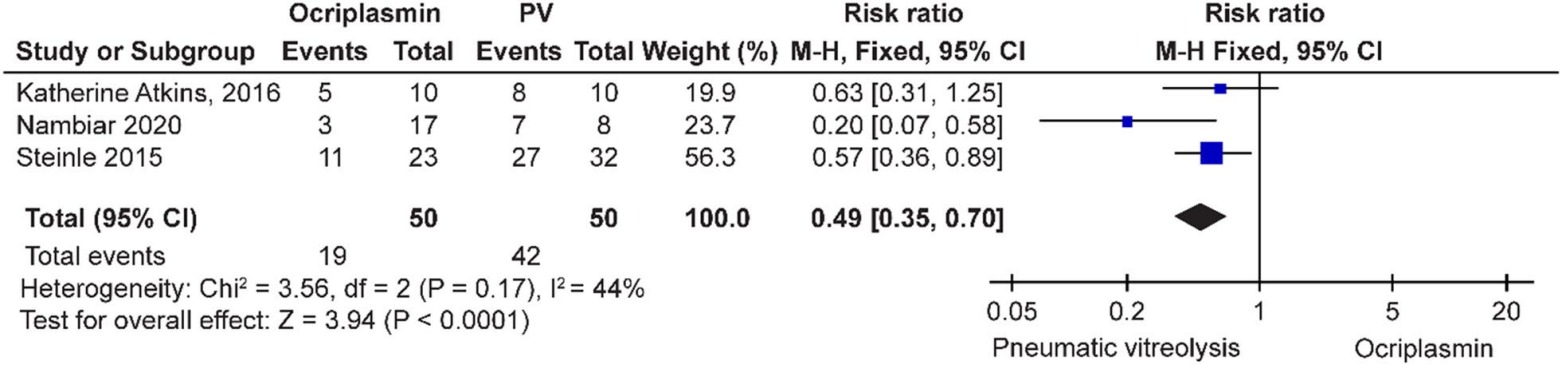
Success rate of ocriplasmin and PV for VMT release

**Fig. 11:**
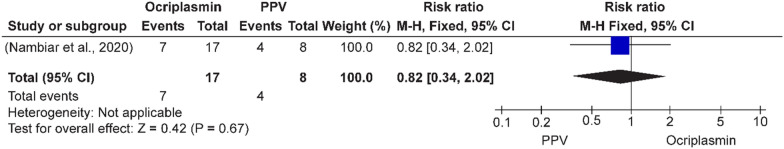
Success rate of ocriplasmin and PPV for MH closure

**Fig. 12:**
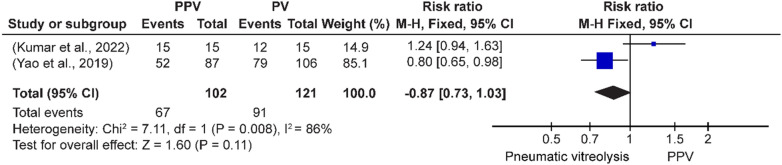
Success rate of PV and PPV for VMT release

**Fig. 13:**
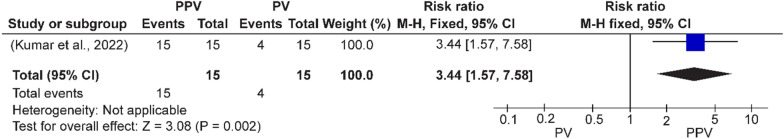
Success rate of PV and PPV for MH closure

Among the three different interventions, PPV had significantly higher rates of VMT release (risk ratio = 0.48, 95% CI 0.38–0.62, *p* = 0.00001) and MH closure (risk ratio = 0.49, 95% CI 0.30–0.81, *p* = 0.006) than ocriplasmin (Figs. 8, 9). The rate of VMT release with PV was significantly higher than that with ocriplasmin (risk ratio = 0.49, 95% CI 0.35–0.70, *p* = 0.0001) (Fig. 10); however, only one study [27] compared the MH closure rate and showed that there was no significant difference (risk ratio = 0.82, 95% CI 0.34, 2.02, *p* = 0.67) between the two groups (Fig. 11). There was no significant difference (risk ratio = 0.87, 95% CI 0.73–1.03, *p* = 0.11) in the VMT release rate between the two groups in terms of therapeutic efficacy (Fig. 12); however, only one study [29] compared the MH closure rate and showed that PPV had a higher rate of MH closure (risk ratio = 3.44, 95% CI 1.57, 7.58, *p* = 0.002), as shown in the funnel plot in Fig. 13.

In addition, other non-comparative studies were also evaluated, and their success rates were calculated manually (Table 3). Approximately 79 different studies were retrieved, and the highest percentage of patients underwent EVL with ocriplasmin treatment for MH closure and VMT release rate. The MH closure rates were 46%, 47.8% and 95%, whereas the VMT release rates after ocriplasmin, PV and PPV treatment were 46%, 47.8% and 100%, respectively. Adverse events and postoperative complications that occurred after these treatments were also documented in these studies.

**Table 3:** The individual study-based data analysis to evaluate the effectiveness of these interventions on macular hole closure rate and VMT release rate

Study	Macular hole			Study	Vitreomacular traction (VMT)		
	Treatment	Events	Total		Treatment	Events	Total
Benz et al. [37]	Ocriplasmin	6	34	Primavera et al. [53]	PV	4	4
Cacciamani et al. [61]	Ocriplasmin	11	23	Čokl et al. [54]	PV	18	29
Cacciamani et al. [62]	Ocriplasmin	11	16	Gruchociak et al. [63]	PV	7	11
Khanani et al. [64]	Ocriplasmin	283	480	Han et al. [41]	PV	10	26
Iuliano et al. [65]	Ocriplasmin	9	16	Arrigo et al. [66]	Ocriplasmin	40	73
Chatziralli et al. [67]	Ocriplasmin	16	24	Bormann et al. [68]	Ocriplasmin	7	10
Wertheimer et al. [69]	Ocriplasmin	13	40	Muqit et al. [46]	Ocriplasmin	4	25
Dugel et al. [40]	Ocriplasmin	55	145	Schumann et al. [49]	Ocriplasmin	28	57
Pirani et al. [70]	Ocriplasmin	9	15	Sharma et al. [50]	Ocriplasmin	21	34
Feng et al. [42]	Ocriplasmin	12	49	Steel et al. [57]	Ocriplasmin	120	295
Meyer et al. [71]	Ocriplasmin	14	22	Makris et al. [72]	Ocriplasmin	15	35
Mastropasqua et al. [73]	Ocriplasmin	7	14	Tadayoni et al. [74]	PPV	15	15
Muqit et al. [46]	Ocriplasmin	4	6	Zandi et al. [58]	Ocriplasmin	34	51
Nudleman et al. [47]	Ocriplasmin	15	36	Seamone et al. [75]	PV	11	20
Quezada-Ruiz et al. [48]	Ocriplasmin	11	23	Anderson et al. [21]	PV	7	9
Schumann et al. [49]	Ocriplasmin	17	25	Baumann et al. [59]	PV	35	47
Stalmans et al. [11]	Ocriplasmin	7	13	Fouad et al. [23]	PV	24	30
Makris et al. [72]	Ocriplasmin	1	3	Özdemir et al. [20]	PV	11	11
Warrow et al. [76]	Ocriplasmin	15	35				
Willekens et al. [52]	Ocriplasmin	27	38				
Itoh et al. [77]	Ocriplasmin	9	19				
Kannan et al. [78]	PPV	77	77				
Cereda et al. [79]	Ocriplasmin	12	15				
Barca et al. [80]	Ocriplasmin	44	74				
Paul et al. [81]	Ocriplasmin	79	167				
Pessoa et al. [82]	Ocriplasmin	27	59				
Wickens et al. [55]	PPV	20	21				
Bormann et al. [68]	Ocriplasmin	4	10				
Pessoa et al. [83]	Ocriplasmin	14	23				
Nambiar et al. [27]	Ocriplasmin	7	17				
Kim et al. [84]	Ocriplasmin	8	19				
Scholz et al. [34]	Ocriplasmin	7	14				
Schumann et al. [85]	Ocriplasmin	45	82				
Sharma et al. [50]	Ocriplasmin	12	32				
Steinle et al. [35]	Ocriplasmin	7	14				
Tadayoni et al. [74]	PPV	4	4				
Zandi et al. [58]	Ocriplasmin	15	21				
Novack et al. [86]	Ocriplasmin	31	74				
Sharma et al. [87]	Ocriplasmin	29	58				
Figueira et al. [88]	Ocriplasmin	47	83				
Tschuppert et al. [89]	Ocriplasmin	5	12				
Reiss et al. [90]	Ocriplasmin	3	10				
Singh et al. [91]	Ocriplasmin	8	17				
Manousaridis et al. [92]	Ocriplasmin	12	35				
Lim et al. [45]	Ocriplasmin	90	200				
Chaudhary et al. [93]	PPV	220	238				
Hejsek et al. [60]	PPV	28	30				
Dihowm et al. [39]	PPV	136	142				
Čokl et al. [54]	PV	1	4				
Han et al. [41]	PV	17	26				
Steel et al. [57]	Ocriplasmin	12	89				
Baumann et al. [59]	PV	4	14				
Özdemir et al. [20]	PV	0	2				
Formula = $\frac{{\mathrm{Number\, of\, Events}}}{{\mathrm{Total \,Number \,of\, eyes}}}\times 100$							
Total number of patients for MH treatment by ocriplasmin = $\frac{1070}{2201}\times 100=48\cdot 6\%$				Total number of patients for MH treatment by ocriplasmin = $\frac{269}{580}\times 100=46\%$			
Total number of patients for MH treatment by PV = $\frac{22}{46}\times 100=47\cdot 8\%$				Total number of patients for MH treatment by PV = $\frac{127}{187}\times 100=68\%$			
Total number of patients for MH treatment by PPV = $\frac{485}{ 512}\times 100=95\%$				Total number of patients for MH treatment by PPV = $\frac{15}{15}\times 100=100\%$			

MH, macular hole; PV, pneumatic vitreolysis; PPV, pars plana vitrectomy; VMT, vitreomacular traction

### Postoperative complications

The postoperative complications reported in different studies are summarized in Table 2. Ocriplasmin treatment resulted in the highest percentage of complications. Complications such as cataracts or lens changes, RTs without RD, intraoperative/postoperative RD, cataract progression/formation, vitreous floaters, photopsia, abnormal color vision test, abnormal ophthalmological examination, and blurred vision, along with their percentages, are listed in Table 2.

## Discussion

The current study compared the functional outcomes and risks of complications associated with three different interventions for VMT syndrome and MH treatment: EVL with ocriplasmin, PV and PPV, and MH treatment. The effectiveness of these different treatments was assessed by conducting a meta-analysis. To the best of our knowledge, this is the first meta-analysis to compare three different interventions for MH and VMT treatment and the first systematic review to analyze the literature regarding the complications that occur as a result of ocriplasmin, PV and PPV as treatments for VMT and MHs. To retrieve relevant literature, several databases and a grey literature searches were conducted. In this meta-analysis, the MH closure rate, VMT release rate and change in VA were the principal outcomes measured. A total of 89 studies were included; among these, 79 were included in the qualitative analysis, and 10 studies were included in the quantitative analysis. The study design, sample size, VMT release rate, MH closure rate, and preoperative and postoperative VA were the characteristics of the included studies that were summarized and documented.

The present study investigated the efficacy of ocriplasmin, PV, and PPV in treating MH and VMT syndrome.  Quantitative analysis revealed no notable variation in the VMT release rate between PV and PPV. However, a significant difference in the MH closure rate was observed, with PPV exhibiting a higher success rate than PV. In the qualitative and individual study-based data analyses, the MH closure rates were 46%, 47.8%, and 95% for ocriplasmin, PV, and PPV, respectively, while the VMT release rates were 46%, 68% and 100% for the same interventions. VA was significantly improved after PPV and PV, but not after ocriplasmin. The findings of this study are consistent with those of previous research, including those reported by Yu et al. [94], showing low VMT release and MH closure rates with ocriplasmin. However, a non-significant reduction in MH size was observed with ocriplasmin treatment. Overall, PPV was found to be the most effective intervention in terms of MH closure and VMT release, whereas PV also showed acceptable results in terms of VMT release compared to ocriplasmin.

In this investigation, not only the functional outcomes but also the associated complications of the different treatments were recorded.  Ocriplasmin treatment was associated with the highest incidence of postoperative complications. This could be attributed to the vitreous liquefaction and protein dissolution at the vitreoretinal interface induced by the ocriplasmin treatment. Floaters and photopsia may occur because of a transient increase in enzymatic activity and vitreoretinal traction, according to Quezada-Ruiz et al. [48]. Previous research has demonstrated that the concentration of ocriplasmin in the vitreous decreases below the quantitative level within seven days after injection;  and hence, most complications are self-limited and improve spontaneously during follow-up [86, 95]. However, severe complications such as cataracts, RD, and RTs may occur as a result of ocriplasmin treatment. Other studies have reported similar results. For instance, a study conducted by Dugel et al. [40] found that ocriplasmin treatment was associated with a higher rate of adverse events than placebo treatment. Similarly, Haller et al. [12] reported that the VMT resolution rate was higher in patients who underwent vitrectomy than in those who underwent ocriplasmin treatment. These findings suggest that ocriplasmin treatment might not be the best option for VMT resolution and that alternative treatment options should be explored.

To mitigate the complications that can arise after ocriplasmin treatment for VMT, safer alternatives such as PPV and PV have been explored. However, the high cost and inherent surgical risks associated with PPV have limited its application to  VMT syndrome. PV, on the other hand, involves the intravitreal injection of a small amount of expansile gas to destabilize the vitreous and promote vitreous liquefaction [18]. This treatment typically requires postural coordination, such as a face-down or drinking bird position. Studies have reported VMT release rates ranging from 56 to 95%, with closure rates of small MHs ranging from 40 to 80% [18, 96]. Despite its effectiveness, potential side effects of PV include MH and RD progression,, which is a concern for both physicians and patients.

The current study analyzing surgery for MH closure has several limitations due to the limited number of available studies, which caused a lack of diversity in the types of studies analyzed. Despite this, all available studies were included in both qualitative and quantitative analyses, regardless of their quality, leading to potential biases. Very few studies were RCTs, and other studies were uncontrolled and potentially prone to confounding factors. The heterogeneity of the studies was substantial due to differences in study populations, inclusion/exclusion criteria, baseline characteristics, study design, clinician's skill, available resources for surgery, adverse event rates, years of research study conduct, and procedures performed. Although the quantitative analysis in the study suggests the need for additional comparative studies to evaluate the efficacy of different techniques for MH closure, very few RCTs are available. The included studies spanned a wide period ranging from 2009 to 2020, and although publication bias and heterogeneity were appropriately controlled, differences in patient indications and baseline characteristics reported in conference abstracts may have influenced the results. Overall, while this study sheds some light on this topic, further research is needed to fully understand the best techniques for MH closure.

## Conclusion

In conclusion, this study aimed to compare the efficacy and safety of three treatment modalities for MH closure and VMT release, including PPV, PV, and EVL with ocriplasmin. The study demonstrated that PPV resulted in a higher MH closure rate of 95% and a VMT release rate of 100%. PV showed lower MH closure and VMT release rates of 47.8% and 68%, respectively, but resulted in a significant reduction in MH size and improvement in vision. Ocriplasmin treatment showed a nonsignificant success rate for both MH closure and VMT release, with values of 46% and 46.3%, respectively, but resulted in a significant improvement in vision. The results of this study suggest that PPV is the most favorable treatment for MH closure and VMT release, with a low incidence of serious complications compared to PV and ocriplasmin. Further research involving large multicenter randomized trials is warranted to confirm the MH closure rates and the effects of ocriplasmin and PV on VA. Additionally, assessing the impact of ocriplasmin treatment on patient quality of life through a literature reviews would be worthwhile.

## Supplementary Information

**Additional file 1.** Literature search strategy.

**Additional file 2.** Modified Downs and Black checklists to assess the quality of the included studies.

## Data Availability

The datasets used in this study are included in the main article. Photographs and figures from this study may be released via a written application to the Photographic Laboratory and Clinical Archives Retina Department of Oftalmologia Integral ABC (Nonprofit Medical and Surgical Organization), Av. Paseo de las Palmas 735 suite 303, Lomas de Chapultepec, Mexico City 11000, Mexico, and the corresponding author upon request.

## Abbreviations

AAO: American Academy of Ophthalmology
ARVO: Association for Research in Vision and Ophthalmology
BCVA: Best-corrected visual acuity
COS: Canadian Ophthalmology Society
CME: Cystoid macular edema
EZ: Ellipsoid zone
EZD: Ellipsoid zone deformity
EVL: Enzymatic vitreolysis
ERM: Epiretinal membrane
FTMH: Full-thickness macular hole
ILM: Internal limiting membrane
IOP: Intraocular pressure
MH: Macular hole
OCT: Optical coherence tomography
ORB: Outer retinal band
PPV: Pars plana vitrectomy
PV: Pneumatic vitreolysis
PVD: Posterior vitreous detachment
PVR: Proliferative vitreoretinopathy
PRISMA: Preferred Reporting Items for Systematic Reviews and Meta-Analysis
SFL: Subfoveal lucency
SRF: Subretinal fluid
SD-OCT: Spectral domain-optical coherence tomography
RCTs: Randomized controlled trials
RD: Retinal detachment
RTs: Retinal tears
SD: Standard deviation
SMD: Standardized mean deviation
SFL: Subfoveal lucency
SRF: Subretinal fluid
VA: Visual acuity
VMA: Vitreomacular adhesion
VMT: Vitreomacular traction
