# Supplementary material for: Pneumatic vitreolysis versus vitrectomy for the treatment of vitreomacular traction syndrome and macular holes: complication analysis and systematic review with meta-analysis of functional outcomes
Source: Int J Retina Vitreous. 2023 May 22;9:33. doi: 10.1186/s40942-023-00472-x (PMC10268451; doi:10.1186/s40942-023-00472-x)
Supplement: Supplementary file 1 — Additional file 1. Literature search strategy. [file 40942_2023_472_MOESM1_ESM.docx]

Medline

| # | Searches | Results |
| --- | --- | --- |
| 1 | Fibrinolysin/ or Peptide fragments/ | 162,903 |
| 2 | (Ocriplasmin or microplasmin or jetrea).mp. [mp=title, abstract, original title, name of substance word, subject heading word, floating sub-heading word, keyword heading word, protocol supplementary concept word, rare disease supplementary concept word, unique identifier, synonyms] | 378 |
| 3 | 1 or 2 | 163,019 |
| 4 | (vitreous detachment) OR (Retinal Detachment) OR (posterior vitreous detachment) OR (macular hole) | 37,386 |
| 5 | (vitreomacular traction syndrome OR VMT OR vitreous detachment OR Retinal Detachment OR posterior vitreous detachment OR macular hole OR Macular hole* OR retinal hole* OR Vitreous Detachment OR one retinal break more retinal breaks OR retinal perforation*).mp.  [mp=title, abstract, original title, name of substance word, subject heading word, floating sub-heading word, keyword heading word, protocol supplementary concept word, rare disease supplementary concept word, unique identifier, synonyms] | 39,341 |
| 6 | 4 or 5 | 39,341 |
| 7 | 3 and 6 | 375 |
| 8 | vitrectomy OR Vitreoretinal Surgery OR pars plana vitrectomy | 25,169 |
| 9 | (vitrectomy OR (Vitreoretinal Surgery) OR (pars plana vitrectomy OR Vitrectom* or vitreoretinal surger*).mp. [mp=title, abstract, original title, name of substance word, subject heading word, floating sub-heading word, keyword heading word, protocol supplementary concept word, rare disease supplementary concept word, unique identifier, synonyms] | 25,169 |
| 10 | 8 or 9 | 25,169 |
| 11 | 3 and 10 | 223 |
| 12 | 6 and 10 | 11,995 |
| 13 | 3 and 6 and 10 | 186 |
| 14 | Sulfur Hexafluoride/ or Intravitreal Injections/ Fluorocarbons | 3,703 |
| 15 | (pneumatic or compressed-air).mp. [mp=title, abstract, original title, name of substance word, subject heading word, floating sub-heading word, keyword heading word, protocol supplementary concept word, rare disease supplementary concept word, unique identifier, synonyms] | 13,472 |
| 16 | 14 or 15 | 17,028 |
| 17 | 3 and 16 | 42 |
| 18 | 6 and 16 | 326 |
| 19 | 10 and 16 | 1,035 |
| 20 | 13 and 16 | 20  *2/5/2023* |

EMBASE

| # | Searches | Results |
| --- | --- | --- |
| 1 | ocriplasmin/ or fibrinolytic agent/ or fibrinolytic factor/ | 28152 |
| 2 | ocriplasmin/ | 539 |
| 3 | (Ocriplasmin or microplasmin or jetrea).mp. [mp=title, abstract, heading word, drug trade name, original title, device manufacturer, drug manufacturer, device trade name, keyword, floating subheading word, candidate term word] | 676 |
| 4 | 1 or 3 | 30910 |
| 5 | 2 or 3 | 30910 |
| 6 | (vitreous detachment) OR (Retinal Detachment) OR (posterior vitreous detachment) OR (macular hole) | 32951 |
| 7 | (vitreomacular traction syndrome OR VMT OR vitreous detachment OR Retinal Detachment OR posterior vitreous detachment OR macular hole OR Macular hole* OR retinal hole* OR Vitreous Detachment OR one retinal break more retinal breaks OR retinal perforation*).mp. [mp=title, abstract, heading word, drug trade name, original title, device manufacturer, drug manufacturer, device trade name, keyword, floating subheading word, candidate term word] | 34344 |
| 8 | 6 or 7 | 34344 |
| 9 | vitrectomy/ or pars plana vitrectomy/ | 34190 |
| 10 | (vitrectomy or Vitreoretinal Surgery or pars plana vitrectomy or Vitrectomy or vitreoretinal surgery).mp. [mp=title, abstract, heading word, drug trade name, original title, device manufacturer, drug manufacturer, device trade name, keyword heading word, floating subheading word, candidate term word] | 34190 |
| 11 | 9 or 10 | 34190 |
| 12 | 4 and 8 | 337 |
| 13 | 5 and 8 | 269 |
| 14 | 4 and 11 | 269 |
| 16 | 8 and 11 | 11962 |
| 17 | 4 AND 8 AND 11 | 179 |
| 18 | Pneumatic.mp. or pneumatic tool/ or intermittent pneumatic compression device/ or pneumatic tourniquet/ | 21427 |
| 19 | Sulfur Hexafluoride/ or Intravitreal Injections/ Fluorocarbons | 10597 |
| 20 | 18 or 19 | 31907 |
| 21 | 4 and 20 | 117 |
| 22 | 8 and 20 | 1260 |
| 23 | 17 and 21 | 23 |
| 24 | 23 and 5 | 23 |

CINAHL

| Search ID# | Search terms | Results |
| --- | --- | --- |
| S1 | ocriplasmin | 94 |
| S2 | Ocriplasmin OR microplasmin OR jetrea | 112 |
| S3 | S1 OR S2 | 140 |
| S4 | (vitreous detachment) OR (Retinal Detachment) OR (posterior vitreous detachment) OR (macular hole) | **3,858** |
| S5 | (vitreomacular traction syndrome OR VMT OR vitreous detachment OR Retinal Detachment OR posterior vitreous detachment OR macular hole OR Macular hole* OR retinal hole* OR Vitreous Detachment OR one retinal break more retinal breaks OR retinal perforation*) | **3986** |
| S6 | S4 OR S5 | 4324 |
| S7 | vitrectomy OR Vitreoretinal Surgery OR pars plana vitrectomy | 2783 |
| S8 | Macular hole surger* OR Vitrectom* OR vitreoretinal surger* OR vitreous resection* OR vitreoretinal surger*  OR pars plana vitrectom* | 3,119 |
| S9 | S7 OR S8 | 3,119 |
| S10 | S3 AND S6 | 67 |
| S11 | S3 AND S9 | 28 |
| S12 | S6 AND S9 | 1496 |
| S13 | S11 AND S9 | 28 |
| S14 | Pneumatic.mp. or pneumatic tool/ or intermittent pneumatic compression device/ or pneumatic tourniquet/ | 372 |
| S15 | Sulfur Hexafluoride/ or Intravitreal Injections/ Fluorocarbons | 132 |
| S16 | S14 OR S15 | 504 |
| S20 | S6 AND S9 AND S16 | 26 |

# Grey Literature

1. **Clinical Trials** [**– https://clinicaltrials.gov/**](https://clinicaltrials.gov/) **(Searched** 2/5/2023 **)**
   1. ocriplasmin OR (macular hole)

9 results

- - 1. Ocriplasmin for Treatment for Symptomatic Vitreomacular Adhesion Including Macular Hole (OASIS)
       1. “The purpose of this study is to evaluate the treatment of symptomatic vitreomacular adhesion / (VMT) including macular hole with ocriplasmin.”
       2. [https://www.clinicaltrials.gov/ct2/show/NCT01429441?term=ocriplasmin+](https://www.clinicaltrials.gov/ct2/show/NCT01429441?term=ocriplasmin+OR+macular+hole+surger*&cond=Macular+Holes&rank=1)

[OR+macular+hole+surger*&cond=Macular+Holes&rank=1](https://www.clinicaltrials.gov/ct2/show/NCT01429441?term=ocriplasmin+OR+macular+hole+surger*&cond=Macular+Holes&rank=1)

- - 1. Intravitreal Microplasmin in Patients Undergoing Surgical Vitrectomy
       1. “The purpose of this trial is to evaluate the safety and preliminary efficacy of different doses and several exposure times of intravitreal microplasmin in the setting of pars plana vitrectomy for vitreomacular traction maculopathy.”
       2. [https://www.clinicaltrials.gov/ct2/show/NCT00123279?term=ocriplasmin+](https://www.clinicaltrials.gov/ct2/show/NCT00123279?term=ocriplasmin+OR+macular+hole+surger*&cond=Macular+Holes&rank=2)

[OR+macular+hole+surger*&cond=Macular+Holes&rank=2](https://www.clinicaltrials.gov/ct2/show/NCT00123279?term=ocriplasmin+OR+macular+hole+surger*&cond=Macular+Holes&rank=2)

- - 1. A Phase III Study of A01016 in Subjects With Symptomatic Vitreomacular

Adhesion

- - - 1. “The purpose of this study is to evaluate the superiority regarding vitreomacular adhesion (VMA) resolution of a single intravitreal injection of A01016 (Ocriplasmin) compared to sham-injection in subjects with symptomatic VMA.”
      2. [https://www.clinicaltrials.gov/ct2/show/NCT01889251?term=ocriplasmin+](https://www.clinicaltrials.gov/ct2/show/NCT01889251?term=ocriplasmin+OR+macular+hole+surger*&cond=Macular+Holes&rank=3)

[OR+macular+hole+surger*&cond=Macular+Holes&rank=3](https://www.clinicaltrials.gov/ct2/show/NCT01889251?term=ocriplasmin+OR+macular+hole+surger*&cond=Macular+Holes&rank=3)

- - 1. Intravitreal Gas for Vitreomacular Adhesion (RELEASE)
       1. “The purpose of this study is to determine the effect of an office-based injection of an intravitreal gas bubble as a treatment for symptomatic vitreomacular adhesion.”
       2. [https://www.clinicaltrials.gov/ct2/show/NCT02001701?term=ocriplasmin+ OR+macular+hole+surger*&cond=Macular+Holes&rank=4](https://www.clinicaltrials.gov/ct2/show/NCT02001701?term=ocriplasmin+OR+macular+hole+surger*&cond=Macular+Holes&rank=4)
    2. Ocriplasmin for Vitreomacular Traction/Symptomatic Vitreomacular Adhesion
       1. The purpose of this study is to observe the anatomical and functional outcomes of ocriplasmin (JETREA™®) over a 6-month period.
    3. A Retrospective Chart Review of Patients Treated With Ocriplasmin for Symptomatic VMA (OZONE)
       1. The purpose of this study is to retrospectively review and further characterize anatomic and symptomatic changes over six months immediately following treatment of symptomatic vitreomacular adhesion (VMA) with JETREA® (ocriplasmin), including incidence, time to onset and resolution of anatomy and symptoms.
    4. Ocriplasmin Research to Better Inform Treatment (ORBIT) (ORBIT)
       1. This is a multicenter, prospective, observational, Phase 4 study that will assess clinical outcomes and safety of JETREA® administered in a real-world setting for the treatment of symptomatic vitreomacular adhesion (VMA) by assessing anatomical and functional outcomes in 1500 patients recruited across approximately 120 USA retina sites.
    5. Assessment of Patients Treated With JETREA® for Vitreomacular Traction
       1. The purpose of this study is to observe the anatomical and functional outcomes of ocriplasmin (JETREA®) over a 6-month follow-up period.
  1. ocriplasmin OR vitreomacular traction syndrome

32 results

- 1. (Macular hole*) AND (Pneumatic.mp. OR Sulfur Hexafluoride/ or Intravitreal Injections/ Fluorocarbons*)

4 results

1. **ProQuest – Dissertations and Theses (Searched** 2/5/2023
   1. (Macular hole* OR retinal perforation*) AND (ocrisplasmin OR microplasmin)

i. 20 results

- 1. noft(Macular hole* OR retinal perforation*) AND noft(ocriplasmin OR macular hole surger*)
     1. 2 results
  2. noft(Macular hole* OR retinal perforation*) AND noft(macular hole surger* OR vitrectom*)
     1. 2 results
  3. noft(Macular hole* OR retinal perforation*) AND noft(ocriplasmin AND macular hole surger*)
     1. 0 results
  4. (Macular hole* OR retinal perforation*) AND (Sulfur Hexafluoride/ OR Intravitreal Injections/ Fluorocarbons OR Pneumatic.mp)
     1. 0 result

1. **Conference Proceeding Searches**

| Conference | Link | Years searched | Search terms | Results/Comments |
| --- | --- | --- | --- | --- |
| ARVO | [https://arv ojournals. org/index. aspx](https://arvojournals.org/index.aspx) | All years | "Meeting abstract" AND (macular hole) AND (macular  hole surgery)  AND  (ocriplasmin) | Searched through meeting  abstracts    7 results    Clinical results of Ocriplasmin versus C3F8 gas for symptomatic Vitreomacular  Traction Syndrome  Katherine Atkins; Simon Taylor  (2016)    Complications of Intravitreal  Ocriplasmin in the Treatment of  Symptomatic Vitreomacular Adhesion  Henry L Feng; Daniel B Roth;  Kunjal K Modi; Howard F Fine;  Harold M Wheatley (2014)    Intravitreal gas injection for the treatment of vitreomacular traction syndrome  Shelley Day; Jose A Martinez;  Peter A Nixon; Mark Levitan;  Clio Armitage Harper (2015)    Foveal Detachment after  Idiopathic Macular Hole  Treatment  Aude Couturier; Elise  Boulanger-Semama; Rabih  Hage; Pascale G Massin; Alain  Gaudric; Ramin Tadayoni  (2015)    Ocriplasmin (Jetrea) and Its  Effects on Photoreceptor  Function  Maribel La Fontaine; Fadi S  Shaya; Kent W Small(2014)    Jetrea (ocriplasmin) as a treatment option for symptomatic vitreomacular traction with or without macular hole (<400 µm) - first clinical experience |

|  |  |  |  | Mathias M Maier; Sophia  Bonse; Christiane Frank;  Nikolaus Feucht; Chris  Lohmann (2014)    Intravitreal Ocriplasmin for  Symptomatic Vitreomacular Adhesion  David Warrow; Auvni Patel;  Joseph Raevis; Michael Lai  (2014)    2/5/2023 |
| --- | --- | --- | --- | --- |
| AAO All Meetings | [https://sec ure.aao.or g/aao/mee tingarchive](https://secure.aao.org/aao/meeting-archive) | “All years available” | Topic: Retina, Vitreous Keywords:  “macular hole*”,  “ocriplasmin”, “macular hole surger*”, “vitrectom*” | No relevant  abstracts/presentations found  2/5/2023 |
| COS | [http://ww w.cossco.ca/cp d/annualmeeting/](http://www.cos-sco.ca/cpd/annual-meeting/) | 2010-2023 | “Macular hole*”,  “ocriplasmin”,  “macular hole surger*”, “vitrectom*” | Searched through abstracts and presentations    Efficacy of preoperative intravitreal injection of gas as an adjunct to pars plana vitrectomy for rhegmatogenous retinal detachment repair Rachel Trussart, Tina Felfeli, Efrem D.  Mandelcorn (2018)  Sedation during vitreoretinal  surgery: Practice patterns in  Canada  ***Jenny Qian****, Scott McCusker,*  *Tania Ligori, Philip Blew,*  *Michael Y.K. Mak, Joshua Barbosa, Varun Chaudhary*  (2018)    Techniques to promote sutureless closure of 23-gauge vitrectomy sclerotomies Vincent Sun, Errol Chan,  Mohab Eldeeb, Ernst Jans Van  Rensburg, John Chen (2018)    Cost analysis of vitrectomy with or without phacoemulsification surgery in the management of macular holes · Walter Andreatta, Kwesi Amassah-  Arthur, Ibrahim Elaroud, Arijit  Mitra (2015)    Interim analysis results from  INJECT: Investigation of JETREA in patients with confirmed vitreomacular traction · Michel Giunta (2015)    Anatomical and visual outcomes following Jetrea treatment for vitreomacular traction – the initial Calgary experience · Michael Fielden, Amin Kherani,  Geoff Williams (2015)    Morbidity and mortality associated with post-operative positioning following vitreoretinal surgery  Munir Iqbal, Lisa Jagan, Jeffrey  Gale, David R. Almeida (2014)    Spontaneous Closure of Traumatic Macular Holes: A Series of 3 Cases.  Micah Luong, Monique Munro,  Micheline C. Deschenes, Feisal  Adatia, Geoff Williams,  Amin Kherani (2013)  2/5/2023 |
