# Supplementary material for: Pneumatic vitreolysis versus vitrectomy for the treatment of vitreomacular traction syndrome and macular holes: complication analysis and systematic review with meta-analysis of functional outcomes
Source: Int J Retina Vitreous. 2023 May 22;9:33. doi: 10.1186/s40942-023-00472-x (PMC10268451; doi:10.1186/s40942-023-00472-x)
Supplement: Supplementary file 2 — Additional file 2. Modified Downs and Black checklists to assess the quality of the included studies. [file 40942_2023_472_MOESM2_ESM.docx]

|  |  | **Reporting** | **External Validity** | **Bias** | **Confounding** |  | **Power** |  |
| --- | --- | --- | --- | --- | --- | --- | --- | --- |
| **Study** | **Year** | **Items 1-10** | **Items 11-13** | **Items 14-20** | **Items 21-26** |  | **Item 27*** | **Total/28** |
| **(abdulmohsen Alreshaid, 2015)** | 2015 | 9 | 3 | 5 | 4 | 1 |  | 22 |
| **(Anderson *et al.*, 2018)** | 2018 | 9 | 2 | 4 | 3 | 1 |  | 18 |
| **(Sharma *et al.*, 2016)** | 2016 | 7 | 3 | 6 | 3 | 1 |  | 18 |
| **(Kumar *et al.*, 2022)** | 2022 | 8 | 2 | 3 | 2 | 1 |  | 16 |
| **(Greven *et al.*, 2016)** | 2016 | 7 | 1 | 4 | 2 | 0 |  | 14 |
| **(Verena R Juncal *et al.*, 2018)** | 2018 | 8 | 1 | 5 | 3 | 0 |  | 17 |
| **(Hejsek *et al.*, 2017)** | 2016 | 8 | 0 | 5 | 2 | 1 |  | 16 |
| **(Steinle *et al.*, 2015)** | 2015 | 8 | 1 | 4 | 3 | 0 |  | 16 |
| **(Atkins *et al.*, 2016)** | 2016 | 7 | 1 | 5 | 2 | 0 |  | 15 |
| **(Seena Nambiar *et al.*, 2020)** | 2020 | 7 | 2 | 5 | 2 | 1 |  | 17 |

Table S1. Modified Downs and Black checklist for assessing the quality of the included studies.
